# Association of Angio-LncRNAs MIAT rs1061540/MALAT1 rs3200401 Molecular Variants with Gensini Score in Coronary Artery Disease Patients Undergoing Angiography

**DOI:** 10.3390/biom12010137

**Published:** 2022-01-15

**Authors:** Mohamed Y. Elwazir, Mohammad H. Hussein, Eman A. Toraih, Essam Al Ageeli, Safya E. Esmaeel, Manal S. Fawzy, Salwa Faisal

**Affiliations:** 1 Department of Cardiology, Faculty of Medicine, Suez Canal University, Ismailia 41522, Egypt; melwazir@med.suez.edu.eg; 2 Division of Endocrine and Oncologic Surgery, Department of Surgery, School of Medicine, Tulane University, New Orleans, LA 70112, USA; mhussein1@tulane.edu; 3 Genetics Unit, Department of Histology and Cell Biology, Faculty of Medicine, Suez Canal University, Ismailia 41522, Egypt; 4 Department of Clinical Biochemistry (Medical Genetics), Faculty of Medicine, Jazan University, Jazan 45142, Saudi Arabia; dr.ageeli@gmail.com; 5 Department of Physiology, Faculty of Medicine, Zagazig University, Zagazig 44519, Egypt; esee2012@yahoo.com; 6 Department of Medical Biochemistry and Molecular Biology, Faculty of Medicine, Suez Canal University, Ismailia 41522, Egypt; dr_salwafaisal@yahoo.com; 7 Department of Biochemistry, Faculty of Medicine, Northern Border University, Arar 1321, Saudi Arabia

**Keywords:** CAD, GATA6-AS, Gensini score, lncRNAs, MALAT1, MIAT, PUNISHER, SENCR

## Abstract

Long non-coding RNAs (lncRNAs) have emerged as essential biomolecules with variable diagnostic and/or prognostic utility in several diseases, including coronary artery disease (CAD). We aimed for the first time to investigate the potential association of five angiogenesis-related lncRNAs (PUNISHER, SENCR, MIAT, MALAT1, and GATA6-AS) variants with CAD susceptibility and/or severity. TaqMan Real-Time genotyping for PUNISHER rs12318065A/C, SENCR rs12420823C/T, MIAT rs1061540C/T, MALAT1 rs3200401T/C, and GATA6-AS1 rs73390820A/G were run on the extracted genomic DNA from 100 unrelated patients with stable CAD undergoing diagnostic coronary angiography and from 100 controls. After adjusting covariates, the studied variants showed no association with disease susceptibility; however, MIAT*T/T genotype was associated with a more severe Gensini score. In contrast, MALAT1*T/C heterozygosity was associated with a lower score. The lipid profile, and to a lesser extent smoking status, male sex, weight, hypertension, and MALAT1 (T > C) (negative correlation), explained the variance between patients/control groups via a principal component analysis. Incorporating the principal components into a logistic regression model to predict CAD yielded a 0.92 AUC. In conclusion: MIAT rs1061540 and MALAT1 rs3200401 variants were associated with CAD severity and Gensini score in the present sample of the Egyptian population. Further large multi-center and functional analyses are needed to confirm the results and identify the underlying molecular mechanisms.

## 1. Introduction

Coronary artery disease (CAD) is a devastating health disorder contributing to high morbidity and mortality rates worldwide [1]. The etiology of CAD is complex and multifactorial, involving environmental and genetic factors [2]. Despite the continuous improvement in anti-ischemic drugs and coronary interventional techniques, CAD patients still suffer from many problems [3]. Therefore, unraveling CAD pathophysiology and developing reliable therapeutic approaches are urgently needed [4].

Long non-coding RNAs have caught the attention of several research groups attempting to define their role in human cells [5]. Numerous biological processes, such as epigenetic modifications, chromatin remodeling, splicing, and cellular differentiation, have been linked to lncRNAs, solidifying their status as critical genetic regulators [6]. Accumulating evidence indicates that lncRNA can play key roles in CAD, including disease diagnosis and/or prognosis [7,8,9,10].

The lncRNA PUNISHER (also known as AGAP2-antisense RNA1) was reported to be significantly upregulated in CAD patients [11]. The SENCR (smooth muscle and endothelial cell-enriched migration/differentiation-associated long non-coding RNA) was highly overexpressed in endothelial cells, smooth muscle cells, and aortic tissue [12].

MIAT (myocardial infarction-associated transcript) gene expression dysregulation could have a potential diagnostic utility in CAD patients as we reported in our previous study [13], and MALAT1 (metastasis-associated lung adenocarcinoma transcript 1) was involved in the proliferation of endothelial cells and associated with the progression of cardiovascular diseases [14]. At the same time, the antisense transcript of GATA6 (GATA6-AS) is involved in endothelial cell (EC) migration and has a repressive effect on angiogenesis [15].

Single nucleotide polymorphisms (SNPs) have been reported to be the most common type of genetic variation associated with population diversity, disease susceptibility/severity, and personalized medicine [16]. SNPs of lncRNAs have been implicated as potential biomarkers in complex disorders such as CAD [17,18]. Based on (1) searching the aforementioned lncRNAs-related SNPs in the dbSNP (www.ncbi.nlm.nih.gov) (last accessed 20 November 2021) for a minor allele frequency (MAF) ≥ 0.1, (2) screening the previous studies that showed evidence of the functional significance of the selected SNPs, and/or (3) no previous studies exploring the impact of these SNPs on the susceptibility and/or severity of CAD patients at least in the Egyptian population, five related SNPs—PUNISHER rs12318065 A/C (chr12: 57726493), SENCR rs12420823 C/T (chr11: 128693497), MIAT rs1061540 C/T (chr22:26666074), MALAT1 rs3200401 T/C (chr11: 65271832), and GATA6-AS1 rs73390820 A/G (chr18: 22168218) according to “Genome Reference Consortium Human Build 38 patch release 13 (GRCh38.p13)”—were included in this study as a preliminary step for future full-scale (including all related SNPs) studies. Hence, we aimed in this study to identify the role of the specified five lncRNAs SNPs as molecular biomarkers of disease risk and/or severity of CAD in a preliminary sample of the Egyptian population.

## 2. Materials and Methods

### 2.1. Study Participants

The current observational case–control study enrolled 100 unrelated Egyptian patients with stable CAD undergoing diagnostic coronary angiography, in addition to 100 controls. The patient group was recruited from the “Cardiology Department, Suez Canal University (SCU) Hospital”, Ismailia, Egypt, between October 2015 and March 2018. The diagnosis of CAD was made through a combination of detailed history taking via a structured interview, clinical examination, resting electrocardiography (ECG), and echocardiography, followed by coronary angiography (CA). Patients with congenital heart disease or vasculitis-related coronary artery disease were excluded. Controls were recruited from healthy unrelated blood donors with no history of cardiovascular problems and a normal resting ECG, regardless of their CAD risk factor profile. They were not subjected to the unnecessary risk of invasive coronary angiography, in compliance with our institutional ethical guidelines. “Declaration of Helsinki” guidelines were followed in this work, and the “Medical Research Ethics Committee” of SCU approved the study. Informed consent was obtained from all participants.

### 2.2. Cardiovascular Disease (CVD) Risk Assessment

Patients were considered to have premature CAD of 55 years for men and 65 years for women [19]. Cardiovascular risk factors were obtained by history. Hypertension was defined as more than two blood pressure measurements over 140/90 mmHg or regular use of anti-hypertensive drugs. Diabetes was defined as “a single random blood sugar ≥ 200 mg/dl, fasting glycemia ≥ 126 mg/dl, or the use of antidiabetic drugs”. Dyslipidemia was diagnosed if at least one of the following conditions were met: “triglycerides (TG) ≥150 mg/dL, total cholesterol (TC) >200 mg/dL, high-density lipoprotein (HDL) <40 mg/dL in males, and <50 mg/dL in females”. Based on the height and weight of participants, the body mass index (BMI) was calculated. Patients were classified as normal (BMI 18.5–24.9), overweight (BMI 25–29.9), or obese (BMI ≥ 30). Patients were considered smokers if they smoked regularly within the previous 12 months. Family history of premature CAD in first-degree male relatives <55 years or females <65 years was recorded.

Several methods were applied for risk assessment, including a “qualitative individual risk factor” approach and a “quantitative estimate (global CAD risk model)”, which are detailed in our previous work [20].

### 2.3. Echocardiography

Two-dimensional echocardiography using a commercially available system (General Electric Healthcare, Vivid 7 Dimension, Vingmed, and Horten, Norway) with a phased array probe (2.5 MHz) was performed (the standard views) on all patients to exclude structural heart disease.

### 2.4. Selective Coronary Angiography

All participants were subjected to angiography following the local hospital protocol (i.e., the modified Seldinger technique) [21,22]. A significant obstruction, defined as “≥50% maximal luminal stenosis in at least one major epicardial coronary artery”, was diagnosed by visual assessment. Vessel score (ranging from 0 to 3) was recorded based on the individual vessels/number of diseased vessels, and accordingly, multivessel disease (MVD) was defined as “≥50% luminal narrowing in more than one major coronary artery” [23].

The modified “Gensini score” was applied to determine CAD extent/severity from coronary angiograms based on “the vessel affected, lesion location, degree of stenosis, the cumulative effect of multiple lesions, and the effect of collaterals” [22,24]. Each vessel score was calculated separately and then summed to yield the total score. A high “Gensini score” was assigned at a cutoff score of 20 [25]. Scores were assigned and interpreted by two independent angiographers who were blinded to the clinical data.

### 2.5. Sample Collection and Laboratory Investigations

For each patient, 6 mL overnight fasting peripheral blood samples were collected: 3 mL in trisodium ethylenediaminetetraacetic acid (1 mg/mL) tubes for genotyping, and 3 mL in serum separator vacutainer tubes. The latter tubes were centrifuged immediately after clotting at 700× *g* for 20 min at room temperature, and the separated serum was aliquoted into Eppendorf (1 mL per aliquot) and stored at –20 °C for later biochemical assay. An enzymatic method was applied for “fasting blood glucose (FBG), total serum cholesterol (TC), high-density lipoprotein cholesterol (HDL-c), and serum triglycerides (TG)” using Hitachi 912 automated chemistry analyzer (Roche Diagnostics Co, Mannheim, Germany). As all participants’ serum TG levels were less than 400 mg/dL, the low-density lipoprotein-cholesterol (LDL-c) value was calculated by Friedewald’s equation [26].

### 2.6. Allelic Discrimination Analysis

DNA was extracted from whole blood via a QIAamp DNA Blood Mini kit (Catalog No. 51104; Qiagen, Hilden, Germany) following the manufacturer’s instructions. NanoDrop ND-1000 (NanoDrop Technologies, Inc., Wilmington, DE, USA) was used to evaluate the extracted DNA concentration/purity. Real-time polymerase chain reaction allelic discrimination was applied in a StepOne™ Real-Time PCR System (Applied Biosystems, Thermo Fisher Scientific, Foster City, CA, USA) using TaqMan assays (Assay IDs: C__30952613_10 (A/C) for PUNISHER rs12318065, C__11783392_10 (C/T) for SENCR rs12420823, C___2467719_10 (C/T) for MIAT rs1061540, C___3246069_10 (T/C) for MALAT1 rs3200401, and C__98039038_10 (A/G) for GATA6-AS1 rs73390820, Applied Biosystems). The details of PCR contents/concentrations and PCR programming were detailed in our previous work [27]. We believe that the no template control (NTC) samples used for every assay that were run on a plate were enough to ensure no contamination. Additionally, we performed an initial run for every assay and selected samples with all three genotypes, including two homozygous and one heterozygous genotype. We used those samples throughout the experiment as controls for every run. Additionally, we used pre-designed TaqMan assays for this study, which allowed us to think that such double specificity, including sequence-specific forward and reverse primers to amplify the polymorphic sequence of interest, together with two TaqMan minor groove binder probes with nonfluorescent quenchers, ensured the success of the reaction and therefore decreased the possibility for false-positive results [28]. A total of 30% of the samples were run in duplicates, with a 100% concordance rate for genotype calls.

### 2.7. Statistical Analysis

The “Statistical Package for the Social Sciences (SPSS)” for Windows software, version 22.0 (IBM Corp., Armonk, NY, USA) and the R version 3.5.1 (R Studio Version 1.2.1335) were applied for data analysis. Genotype analysis and Hardy–Weinberg equilibrium (HWE) calculation in patients and controls were estimated using SNPStats software (https://www.snpstats.net/) (last accessed 14 April 2021). Adjusted odds ratio (OR) and 95% confidence interval (CI) were calculated for each genetic association model (allelic model, homozygote/heterozygote comparison, dominant, and recessive models) [29]. Categorical variables were quoted as frequencies and percentages and were compared using the chi-square (χ^2^) or Fisher’s exact tests where appropriate. Continuous data are presented as mean ± standard deviation (SD) and were compared using Student’s *t*-test if the data distribution was parametric. Otherwise, Mann–Whitney U (MW) and Kruskal–Wallis tests were applied. Spearman’s rank correlation coefficient was run for correlations analysis. A two-tailed *p*-value less than 0.05 was considered statistically significant. Stepwise logistic regression was performed to detect independent predictors of CAD. The ggplot2 package was used for multivariate analysis.

## 3. Results

### 3.1. Clinical Characteristics of the Study Population

A total of 100 patients and 100 controls were included in the study. Study participants’ baseline characteristics are shown in Table 1. The mean age was 56 years, with no significant difference between groups. The study group showed a stronger male predominance than the control group (64% vs. 52%). The prevalence of medical comorbidities was not significantly different between groups. However, many more smokers were present in the study group (70% vs. 15% in the control group). Average weight and BMI were significantly higher in the study group (84 and 30 vs. 77 and 28 kg/m^2^, respectively). All lipid profile parameters were significantly different between groups, with mean HDL being higher in the control group (50 vs. 38 mg/dL) and LDL, total cholesterol, and triglycerides being higher in the study group (145, 221, and 182 mg/dL vs. 77, 168, and 95 mg/dL, respectively).

### 3.2. Genotype Analysis

Genotype frequencies followed the HWE in the control group, except in the case of the MALAT1 variant. No significant difference in genotype or allele frequencies was observed between patients and controls (Table 2).

**Figure 1:**
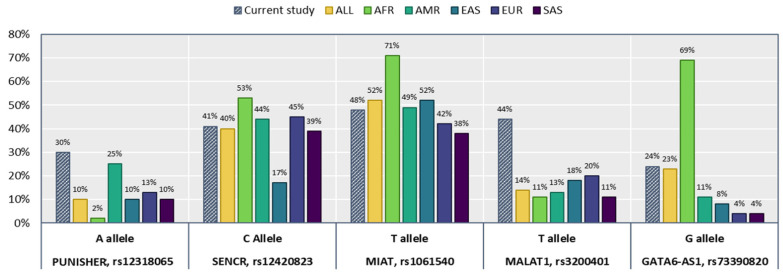
Minor allele frequency of the study lncRNA gene variants in the current study compared with 1000 Genomes Project Phase 3 (https://www.internationalgenome.org/) (last accessed 17 April 2021). AFR, Africa; AMR, America; EAS, East Asia; EUR, Europe; SAS, South Asia.

Minor allele frequency (MAF) for each SNP was 30% (rs12318065*A), 41% (rs12420823*C), 48% (rs1061540*T), 44% (rs3200401*T), and 24% (rs73390820*G) (Figure 1). After adjustment of covariates, no association was found with disease susceptibility (Table 3). Consistently, multiple combinations of genotypes did not render a differential frequency pattern (Table 4).

### 3.3. Association of LncRNA Variants and Disease Outcomes

Patients with MIAT*T/T genotype were associated with a more severe Gensini score. In contrast, MALAT1*T/C heterozygosity was associated with a lower Gensini score (Table 5).

### 3.4. Multivariate Analysis

**Figure 2:**
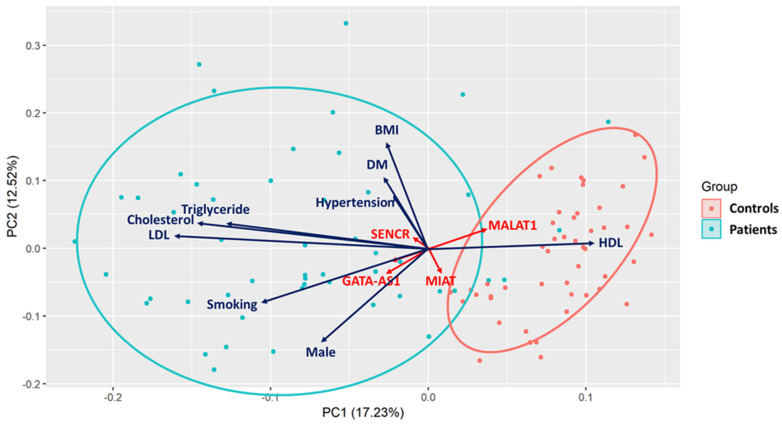
Multivariate analysis for combined environmental variables and the studied genetic variants. Arrow length indicates the strength of the effect, while direction indicates the vector. The red-colored circle (the right one) encloses the control group, while the teal-colored circle (the left one) encloses the patient group. N.B. All the studied (genetic and non- genetic) variables were included in a preliminary principal component analysis, then the significant variables were only shown.

Principal component analysis (PCA) was used to determine the effect of various variables on overall variance in the groups. A PCA biplot is shown in Figure 2. Significant variables explaining most of the variance were the lipid profile parameters—namely, LDL, total cholesterol, triglycerides, and HDL. These variables effectively separated patients into study and controls (i.e., predicting CAD), with HDL inversely associated with CAD and the others positively associated. Other variables that were also predictive but not as strongly included smoking, male sex, weight, and hypertension (positive correlation) and MALAT1 (T >C ) (negative correlation). Incorporating the first two principal components (i.e., PC1 and PC2) into a logistic regression model to predict CAD yielded a 0.92 area under the curve.

## 4. Discussion

Given that angio-lncRNAs are essential regulators of angiogenesis and that growing studies have shed light on their critical role in atherosclerosis and CAD [30], we genotyped for the first time five variants of angiogenesis-related lncRNAs (PUNISHER, SENCR, MIAT, MALAT1, and GATA) to investigate their putative association with CAD risk and/or severity. Our study demonstrated variability in genotype analysis, disease risk, and Gensini score.

The study demonstrated that the MIAT rs1061540 T/T genotype was associated with a more severe Gensini score under codominant and recessive models, although no association with disease susceptibility was observed. In agreement with our outcome, TT homozygosity of the same MIAT variant was associated with a more advanced grade of diabetic retinopathy compared with CC and TC genotypes [27]. Ishii et al. conducted a large-scale association study and reported that six MIAT SNPs (i.e., rs2331291, rs2301523, exon 3 (8813), and exon 5 (11093, 11741, and 12311)) were associated with myocardial infarction (MI) risk; however, the current study polymorphism was not found to be a risk variant [31], which could support in part our finding of inability to identify an association of this variant with the disease risk.

Recently, Ma et al. investigated ten polymorphisms of MIAT promoters and found that two SNPs (rs5752375T/C and rs9608515T/C) were associated with MI, with the TT genotype being a risk factor compared with the CC genotype, potentiating the role of MIAT SNPs in CAD development [32].

It is worth noting that the functional role of MIAT polymorphisms might be related to gene expression alteration. A minor allele at exon 5 11,741 G>A SNP of MIAT increased its transcription compared with major allele G. Additionally, rs5752375 and rs9608515 polymorphisms of MIAT promotor might influence binding of the transcription factors, causing it to lose some and interact with others [31,32]. MIAT was highly expressed in the atheromatous plaques [33] and the peripheral blood of CAD patients. Its expression level was associated with the severity of CAD in terms of the Gensini score [13]. Similar findings were reported in acute myocardial infarction (AMI), including upregulation and correlation with the degree of myocardial damage [34], potentiating the implication of MIAT in ischemic heart diseases.

The deregulation of MIAT, a proatherogenic lncRNA, may contribute to CAD through several epigenetic mechanisms [35]. MIAT, being a competitive endogenous RNA (ceRNA), interacts with miR-150-5p, releasing its inhibitory effect on VEGF (causing endothelial cell dysfunction and pathological angiogenesis) [36]. It targets the miR-145 with upregulation of the PI3K/Akt/Bcl-2 signaling pathway and consequently improves the viability and inhibits the programmed cell death of vascular smooth muscle cells [37]. It sponges miR-149-5p, increasing the expression of CD47 (antiphagocytic), leading to plaque vulnerability [38]. Additionally, MIAT stimulates cellular proliferation through the miR-181b/STAT3 signaling pathway in an atherosclerosis cell model [39].

In contrast with MIAT, the present work reports that MALAT1 (T/C) heterozygosity was associated with a low Gensini score and could be considered a protective factor for CAD, according to multivariate analysis. It is worth noting that on analyzing the study MALAT1 variant against the HWE in the control group, it showed deviation from HWE due to an increase in the wild/mutant homozygous genotypes compared with the expected values (38/34 vs. 27/23, respectively). Although it is difficult to speculate the main reason for this, some of the possible causes of population differences shown in the study are selection (the study groups were selected from the hospital), small population size, population stratification, and genetic drift [40,41]. A genotyping error should also be considered as “in many genotyping platforms, calling heterozygotic individuals is more challenging than homozygotic individuals, and a higher rate of missing individuals for this genotype can distort HWE” [42].

Consistent with our findings, the MALAT1 rs3200401 (T/C) genotype was protective against diabetic retinopathy and associated with decreased disease susceptibility [27]. Moreover, Wang et al. conveyed that MALAT1 rs3200401 CT or CT + TT genotype was associated with more prolonged survival and lower mortality than the CC genotype in advanced lung cancer [43]. On the other hand, Wang and his colleagues reported no association between the MALAT1 (rs3200401) variant and CAD risk. Still, they demonstrated that patients with CT/TT genotypes had lower total cholesterol levels [14]. In acute myocardial infarction, Li et al. recently demonstrated no association between the MALAT1 variant and disease risk [44]. MALAT1 (rs3200401) was investigated in another atherosclerotic disease, such as ischemic stroke and other cardiac diseases such as congenital heart disease, and no association with disease risk or severity was noted [45,46].

Of note, several studies have uncovered an association between CAD and MALAT1. It was significantly upregulated in the blood of CAD patients of different clinical phenotypes and associated with disease severity [13,46,47,48]. Moreover, recent research has indicated that MALAT1 could not only be a helpful diagnostic cardiac biomarker but also valuable for the prediction of in-stent restenosis [49].

MALAT1 can contribute to CAD in several ways. It potentiates endothelial growth and proliferation through modulating the expression of cell cycle regulatory genes [50] and upregulating both VEGF (by miR-145 sponge) and fibroblast growth factor 2 [51]. MALAT1 causes defective endothelial cell autophagy (by upregulating the mTOR signaling pathway through the MALAT/miR-15b-5p/MAPK1 signal axis) [52] and apoptosis (by inhibiting the caspase activity through PI3K/Akt pathway) [14]. Several in vitro/in vivo studies have shown evidence that MALAT1 promotes glucose-induced inflammation of vascular endothelial cells, causing EC injury and dysfunction through various molecular mechanisms (MALAT1/miR-361-3p/SOCS3 axis, MALAT1/miR-22/NLRP3, MALAT1/serum amyloid antigen/TNF, IL6 [53,54,55,56]. Moreover, MALAT 1 augments lipid uptake by macrophage and subsequently foam cell formation by enhancing CD36 transcription through a β-catenin-dependent mechanism [51]. In addition to its effect on EC, MALAT1 promotes vascular smooth muscle proliferation and migration by miR-124-3p sponge [57].

Interestingly, it has been suggested, using the lncRNA SNP database (10.1093/nar/ gkx1004) [1], that the C/T variant of rs3200401 causes 1.62 kcal/mol minimal free energy change that could alter the structure of MALAT1, leading to weakened interaction with its binding protein, serine/arginine-rich splicing factor 2 (SRSF2 and loss of miRNA–MALAT1 binding (as hsa-miR–1324 miRNA)) [58]. Loss of SRSF2 binding could downregulate the phosphorylation of SRFS2, being responsible for alternative splicing of several pre-mRNAs (such as VEGFA/VEGFR). Collectively, it is biologically possible to indicate that deregulation of the molecular mechanisms by MALAT1 (rs3200401C/T) SNP may influence the stability and molecular sponging function of MALAT1, leading to a less pathophysiological derangement.

Moreover, the MALAT1 rs3200401 variant was shown to cause alterations in two regulatory motifs—namely, GATA-binding protein (GATA) and glucocorticoid nuclear receptor GR [2]. The transcription factor GATA2 was identified as contributing to the etiology of CAD [59]. On the other hand, the glucocorticoid receptor is a member of the nuclear receptor family that controls many distinct gene networks, governing various aspects of development, metabolism, inflammation, and the stress response, as well as other vital biological processes in the cardiovascular system [60].

According to HaploReg v4.1 (https://pubs.broadinstitute.org/mammals/haploreg/haploreg.php) (last accessed 5 November 2021), MALAT1 rs3200401 was found to be in linkage disequilibrium with such other nearby SNPs as rs11227206 (7.7 kb 5′ prime end of MALAT1), rs4102217 (1.3 kb 5′ prime end of MALAT1), and rs10896012 (4.5 kb at the 3′ prime end of MALAT1), which might be an additional candidate SNP associated with CAD pathogenesis.

Regarding SENCR (rs12420823 C/T), the current study did not show a significant difference in allele or genotype frequency between cases and controls. Additionally, no association with a disease risk was noted. Mohammad et al. investigated this SENCR polymorphism in DR, and it was associated only with better pre-treatment best-corrected visual acuity levels. Shahmoradi et al. conducted a study of another SENCR polymorphism (rs555172 A/G) in CAD patients, and no association was found, but the frequency of GG genotype was higher in females compared with male patients [17]. SENCR, a vascular-enriched lncRNA, is abundantly expressed in vascular endothelial cells (ECs) and smooth muscle cells (SMCs). It correlates with the expression of the Friend leukemia integration virus 1 (FLI1) gene, an essential regulator of endothelial function. SENCR is involved in the regulation of migration and differentiation of SMCs and ECs by controlling the expression of pre-migratory genes, e.g., PTN (pleiotrophin) and MDK (midkine) and contractile genes such as MYOCD (myocardin) and ACTA2 (actin alpha 2) [12,61,62]. Moreover, it is induced by laminar shear stress and plays a crucial role in regulating EC membrane integrity and permeability. This role has been evidenced to be mediated by binding to cytoskeletal-associated protein 4 (CKAP4), leading to proper localization of CDH5 at the endothelial cell adherens junction, stabilizing it [63].

Regarding the GATA6-AS1 (rs73390820 A/G) variant, its related genotypes did not show significant association with either disease risk or severity. GATA6-AS1 is hypoxia-induced lncRNA enriched in endothelial cells. It controls EC migration and has a repressive effect on angiogenesis. GATA6-AS silencing diminishes endothelial–mesenchymal transition (being recognized as the causal contributor of various cardiovascular pathologies) in vitro and promotes angiogenesis in vivo. It epigenetically regulates endothelial gene expression through interacting with nuclear lysyl oxidase-like2 (LOXL2), impairing its function as a deaminase of trimethylated lysine 4 of histone H3 (H3K4me3), the chromatin signature for transcriptional activation [15]. Notably, GATA6-AS1 was evidenced as an upstream inhibitor of taurine upregulated gene 1 (TUG1) [64], which is known to play a crucial role in promoting atherosclerosis [65]. Additionally, GATA6-AS1 has been found to promote cardiomyocyte differentiation from human pluripotent stem cells (hPSCs) [66]. Such findings raise the possibility of a cardio-protective role of GATA6-AS that seems in line with our results and for further confirmation in future studies.

Our findings also did not show a significant association with the disease risk/severity regarding PUNISHER rs12318065 A/C variant. PUNISHER is one of the essential angio-lncRNAs specifically expressed in endothelial cells differentiated from human pluripotent stem cells. PUNISHER inhibition has been associated with significant vascular defects such as branching defects and malformed blood vessels, as well as impaired EC function. These findings were associated with the downregulation of mitotic and cell division genes and upregulation of genes involved in cell adhesion and extracellular interaction [67,68]. Taken together, these findings may support the vital role of PUNISHER in endothelial function and vascularity and support our results regarding the negative association with CAD risk or severity. Additional work is warranted to confirm our results.

As CAD is a complex disease involving multiple genes and demographic/environmental factors, such as age, sex, lipid profile, associated comorbidities, etc., the development and severity of CAD cannot only be explained by gene variations and polymorphisms [69]. Additionally, the genetic variants often exert their effect in “a cell-type-specific and context-dependent manner” [18]. Collectively, this could partly explain the conflicting findings (if present) between the present study and previous ones. Other contributing elements such as ethnicity, geographical factors, and different sample size and methodology of the various studies should also be considered.

Although this study is the first to uncover the impact of five angio-lncRNAs polymorphisms on CAD susceptibility, some limitations should be considered. First, our study was a hospital-based case–control study, making it challenging to avoid selection bias. Second, the relatively small sample size may limit detection of small effect sizes of some study variants. Third, the limited selected study variants with MAF ≥ 0.10 were included in the study to achieve adequate statistical power. Fourth, the genotype distribution of the MALAT1 variant was inconsistent with HWE, which could raise the potential of false-positive association due to genotyping errors (if any) that warrant further validation by another method (e.g., by direct sequencing of some selected samples). Therefore, it is recommended to replicate the work in multi-center, larger-scale studies in different ethnic groups. Moreover, including other SNPs related to the studied lncRNAs and other angio-related lncRNAs (to avoid the subjective selection bias for the type of lncRNA variants), together with functional analysis, will be helpful.

## 5. Conclusions

This study shows that MIAT rs1061540 and MALAT1 rs3200401 variants are associated with CAD severity and Gensini score. Further large multi-center and functional analyses are needed to confirm the association’s significance and identify the underlying molecular mechanism of variant action.

## Figures and Tables

**Table 1:** Baseline characteristics of the study population.

Characteristics	Controls (n = 100)	Patients (n = 100)	*p*-Value
Demographic data			
Age, years			
Mean ± SD	56 ± 9.0	56.0 ± 9.0	0.876
Sex			
Male	52 (52.0)	64 (64.0)	**0.023**
Weight, kg	77 ± 6.0	84 ± 11	0.118
Height, cm	165 ± 7.0	168 ± 6.6	0.713
BMI, kg/m^2^	28 ± 2.0	30 ± 0.5	**0.038**
Obesity	2 (2.0)	44 (44.0)	**0.001**
Family history of CVD	36 (36.0)	22 (22.0)	0.123
Smoking	16 (16.0)	70 (70.0)	**<0.001**
**Clinical data**			
DM	28 (28.0)	44 (44.0)	0.096
HTN	38 (38.0)	54 (54.0)	0.108
Premature CAD	---	80 (80.0)	NA
Previous events	---	80 (80.0)	NA
Stroke	---	2 (2.0)	NA
Aneurysms	---	2 (2.0)	NA
**Echocardiography**			
Dias BP, mmHg	---	82 ± 14	NA
Pulse, bpm	---	87 ± 13	NA
EDD	---	52 ± 7.0	NA
ESD	---	38 ± 6.0	NA
PW	---	9 ± 2.0	NA
SW	---	9 ± 2.0	NA
EF	---	55 ± 13	NA
**Angiography**			
Gensini score	---	38 ± 43	NA
Vessel score	---	2.0 ± 2.0	NA
**Laboratory data**			
HDL-c	50 ± 7.0	38 ± 13	**<0.001**
LDL-c	77 ± 12	145 ± 49	**<0.001**
TC	168 ± 18	221 ± 50	**<0.001**
TG	95 ± 35	182 ± 72	**<0.001**
FBS	---	151 ± 67	NA

Data are shown as mean ± standard deviation (SD) or numbers (percentage). CVD, cardiovascular disease; DM, diabetes mellitus; HTN, hypertension; CAD, coronary artery disease; previous events, previous acute coronary ischemic events; Dias Bp, diastolic blood pressure; EDD, left ventricular end—diastolic diameter; ESD, left ventricular end—systolic dimension; PW, posterior wall thickness; SW, septal wall thickness; EF, ejection fraction; HDL-c, high-density lipoprotein-cholesterol; LDL-c, low-density lipoprotein-cholesterol; TC, total cholesterol; TG, triglycerides; FBS, fasting blood sugar; NA, not applicable. Gensini/vessel scores were calculated to assess disease severity. *p*-values were calculated by using Fisher’s exact test and Student’s *t*-tests. The bold values indicate statistically significant *p* < 0.05.

**Table 2:** Genotype and allele frequencies of the study lncRNA variants.

Gene	Frequency	Variant	All		Controls		Patients		*p*-Value
			n	Proportion	n	Proportion	n	Proportion	
**PUNISHER** (AGAP2-AS1) **rs12318065**	**Genotype frequency**	A/A	26	0.13	10	0.1	16	0.16	0.15
		C/A	70	0.35	44	0.44	26	0.26	
		C/C	104	0.52	46	0.46	58	0.58	
	P _HWE_				1.00				
	**Allele frequency**	C	278	0.7	136	0.68	142	0.71	0.64
		A	122	0.3	64	0.32	58	0.29	
**SENCR** (FLI1) **rs12420823**	**Genotype frequency**	C/C	24	0.12	12	0.12	12	0.12	0.66
		T/C	116	0.58	62	0.62	54	0.54	
		T/T	60	0.3	26	0.26	34	0.34	
	P _HWE_				0.08				
	**Allele frequency**	T	236	0.59	114	0.57	122	0.61	0.56
		C	164	0.41	86	0.43	78	0.39	
**MIAT** **rs1061540**	**Genotype frequency**	C/C	70	0.35	30	0.3	40	0.4	0.47
		C/T	66	0.33	38	0.38	28	0.28	
		T/T	64	0.32	32	0.32	32	0.32	
	P _HWE_				0.09				
	**Allele frequency**	C	206	0.52	98	0.49	108	0.54	0.47
		T	194	0.48	102	0.51	92	0.46	
**MALAT1** **rs3200401**	**Genotype frequency**	C/C	92	0.46	38	0.38	54	0.54	0.15
		C/T	42	0.21	28	0.28	14	0.14	
		T/T	66	0.33	34	0.34	32	0.32	
	P _HWE_				0.001				
	**Allele frequency**	C	226	0.56	104	0.52	122	0.61	0.19
		T	174	0.44	96	0.48	78	0.39	
**GATA6-AS1** **rs73390820**	**Genotype frequency**	A/A	114	0.57	58	0.58	56	0.56	0.62
		A/G	74	0.37	34	0.34	40	0.4	
		G/G	12	0.06	8	0.08	4	0.04	
	P _HWE_				0.47				
	**Allele frequency**	A	302	0.76	150	0.75	152	0.76	0.86
		G	98	0.24	50	0.25	48	0.24	

A chi-square test was applied. N, number; P _HWE_, *p*-value of “Hardy–Weinberg equilibrium” calculation. Significance was set at *p* < 0.05.

**Table 3:** Genetic association models of lncRNA variants and disease risk.

Gene	Model	Genotype	Controls	Patients	Crude OR (95% CI)	*p*-Value	Adjusted OR (95% CI)	*p*-Value
**PUNISHER**	Codominant	C/C	46 (46%)	58 (58%)	1.00	0.15	1.00	0.39
		A/C	44 (44%)	26 (26%)	0.47 (0.19–1.13)		0.47 (0.16–1.41)	
		A/A	10 (10%)	16 (16%)	1.27 (0.37–4.40)		0.80 (0.17–3.73)	
	Dominant	C/C	46 (46%)	58 (58%)	1.00	0.23	1.00	0.23
		A/C–A/A	54 (54%)	42 (42%)	0.62 (0.28–1.36)		0.54 (0.20–1.49)	
	Recessive	C/C–A/C	90 (90%)	84 (84%)	1.00	0.37	1.00	0.90
		A/A	10 (10%)	16 (16%)	1.71 (0.52–5.66)		1.10 (0.26–4.73)	
	Log-additive	---	---	---	0.89 (0.51–1.55)	0.67	0.76 (0.37–1.53)	0.44
**SENCR**	Codominant	T/T	26 (26%)	34 (34%)	1.00	0.67	1.00	0.77
		C/T	62 (62%)	54 (54%)	0.67 (0.27–1.62)		0.83 (0.26–2.59)	
		C/C	12 (12%)	12 (12%)	0.76 (0.20–2.93)		1.49 (0.27–8.30)	
	Dominant	T/T	26 (26%)	34 (34%)	1.00	0.38	1.00	0.89
		C/T-C/C	74 (74%)	66 (66%)	0.68 (0.29–1.61)		0.93 (0.31–2.76)	
	Recessive	T/T-C/T	88 (88%)	88 (88%)	1.00	1.00	1.00	0.52
		C/C	12 (12%)	12 (12%)	1.00 (0.30–3.34)		1.68 (0.35–8.08)	
	Log-additive	---	---	---	0.81 (0.43–1.53)	0.52	1.10 (0.49–2.45)	0.82
**MIAT**	Codominant	C/C	30 (30%)	40 (40%)	1.00	0.48	1.00	0.25
		C/T	38 (38%)	28 (28%)	0.55 (0.21–1.45)		0.54 (0.16–1.83)	
		T/T	32 (32%)	32 (32%)	0.75 (0.29–1.97)		0.35 (0.10–1.28)	
	Dominant	C/C	30 (30%)	40 (40%)	1.00	0.29	1.00	0.12
		C/T-T/T	70 (70%)	60 (60%)	0.64 (0.28–1.47)		0.44 (0.15–1.27)	
	Recessive	C/C-C/T	68 (68%)	68 (68%)	1.00	1.00	1.00	0.18
		T/T	32 (32%)	32 (32%)	1.00 (0.43–2.32)		0.46 (0.14–1.48)	
	Log-additive	---	---	---	0.86 (0.53–1.39)	0.54	0.59 (0.31–1.12)	0.09
**MALAT1**	Codominant	C/C	38 (38%)	54 (54%)	1.00	0.15	1.00	0.39
		T/C	28 (28%)	14 (14%)	0.35 (0.12–1.04)		0.43 (0.11–1.68)	
		T/T	34 (34%)	32 (32%)	0.66 (0.27–1.63)		0.53 (0.16–1.73)	
	Dominant	C/C	38 (38%)	54 (54%)	1.00	0.11	1.00	0.18
		T/C-T/T	62 (62%)	46 (46%)	0.52 (0.24–1.16)		0.49 (0.17–1.40)	
	Recessive	C/C-T/C	66 (66%)	68 (68%)	1.00	0.83	1.00	0.53
		T/T	34 (34%)	32 (32%)	0.91 (0.40–2.10)		0.71 (0.24–2.08)	
	Log-additive	---	---	---	0.79 (0.51–1.24)	0.31	0.72 (0.40–1.30)	0.27
**GATA6-AS1**	Codominant	A/A	58 (58%)	56 (56%)	1.00	0.62	1.00	0.84
		A/G	34 (34%)	40 (40%)	1.22 (0.53–2.79)		1.01 (0.36–2.82)	
		G/G	8 (8%)	4 (4%)	0.52 (0.09–3.06)		0.45 (0.03–7.02)	
	Dominant	A/A	58 (58%)	56 (56%)	1.00	0.84	1.00	0.90
		A/G-G/G	42 (42%)	44 (44%)	1.09 (0.49–2.40)		0.94 (0.35–2.56)	
	Recessive	A/A-A/G	92 (92%)	96 (96%)	1.00	0.40	1.00	0.55
		G/G	8 (8%)	4 (4%)	0.48 (0.08–2.74)		0.45 (0.03–6.78)	
	Log-additive	---	---	---	0.95 (0.50–1.81)	0.87	0.88 (0.37–2.08)	0.76

Data are shown as the number (%). OR (95% CI), odds ratio (95% confidence interval). Adjusted covariates by age and sex. A chi-square test was used. Significance was set at *p* < 0.05.

**Table 4:** Combined genotype association with disease risk.

	PUNISHER	SENCR	MIAT	MALAT1	GATA6-AS1	Total	Controls	Patients	Cumulative Frequency
1	C	T	C	T	A	0.0917	0.1292	0.0578	0.0917
2	C	C	T	C	A	0.0871	0.0803	0.0923	0.1788
3	C	T	T	C	G	0.0817	0.0782	0.0687	0.2605
4	C	T	T	T	A	0.0724	0.1059	0.0685	0.3329
5	C	C	C	C	A	0.072	0.0434	0.0609	0.4049
6	C	T	C	C	A	0.0711	0.0508	0.1244	0.476
7	A	C	C	C	A	0.0645	0.1095	NA	0.5405
8	A	T	T	T	A	0.0615	0.0376	0.05	0.602
9	A	T	C	C	A	0.0457	0.0493	0.0626	0.6477
10	C	C	C	T	A	0.0377	0.0206	0.0614	0.6854
11	C	C	T	T	G	0.0376	0.0341	0.0285	0.723
12	C	C	T	T	A	0.033	0.0409	0.013	0.756
13	A	T	T	C	A	0.033	1e-04	0.0777	0.789
14	A	T	C	C	G	0.0264	0.0224	0.0231	0.8154
15	A	T	C	T	A	0.0235	NA		

Global haplotype association *p*-value: 0.003.

**Table 5:** Association of lncRNA variants with Gensini score during angiography.

Gene	Model	Genotypes	n	Gensini Score Mean (SEM)	Difference (95% CI)	*p*-Value
**PUNISHER**	Codominant	C/C	58	36.86 (7.69)	*Reference*	0.38
		A/C	26	49.92 (13.84)	14.20 (−13.18, 41.58)	
		A/A	16	23.12 (12.31)	−11.08 (−43.92, 21.75)	
	Dominant	C/C	58	36.86 (7.69)	*Reference*	0.71
		A/C-A/A	42	39.71 (9.99)	4.61 (−19.13, 28.35)	
	Recessive	C/C-A/C	84	40.9 (6.79)	*Reference*	0.34
		A/A	16	23.12 (12.31)	−15.51 (−47.23, 16.21)	
	Log-additive	---	---	---	−1.70 (−17.34, 13.93)	0.83
**SENCR**	Codominant	T/T	34	40.24 (11.74)	*Reference*	0.75
		C/T	54	39.56 (7.89)	−0.70 (−26.96, 25.55)	
		C/C	12	25.17 (15.95)	−14.39 (−53.90, 25.12)	
	Dominant	T/T	34	40.24 (11.74)	*Reference*	0.79
		C/T-C/C	66	36.94 (7.04)	−3.39 (−28.44, 21.65)	
	Recessive	T/T-C/T	88	39.82 (6.55)	*Reference*	0.45
	C/C		12	25.17 (15.95)	−13.97 (−49.91, 21.96)	
	Log-additive	---	---	---	−5.39 (−23.64, 12.85)	0.75
**MIAT**	Codominant	C/C	40	28.2 (8.65)	*Reference*	0.06
		C/T	28	31.86 (9.52)	5.47 (−22.34, 33.27)	
		T/T	32	55.81 (12.51)	**31.88 (5.29, 58.47)**	
	Dominant	C/C	40	28.2 (8.65)	*Reference*	0.11
		C/T-T/T	60	44.63 (8.19)	19.70 (−3.79, 43.18)	
	Recessive	C/C-C/T	68	29.71 (6.33)	*Reference*	0.019
		T/T	32	55.81 (12.51)	**29.64 (5.83, 53.46)**	
	Log-additive	---	---	---	**15.63 (2.40–28.86)**	0.025
**MALAT1**	Codominant	C/C	54	36.67 (8.64)	*Reference*	0.044
		T/C	14	16.29 (8.33)	**−35.10 (−69.90–−0.30)**	
		T/T	32	49.94 (11)	13.68 (−11.54–38.90)	
	Dominant	C/C	54	36.67 (8.64)	*Reference*	0.97
		T/C-T/T	46	39.7 (8.6)	−0.43 (−24.65–23.79)	
	Recessive	C/C-T/C	68	32.47 (7.17)	*Reference*	0.11
		T/T	32	49.94 (11)	20.68 (−4.32–45.68)	
	Log-additive	---	---	---	5.42 (−7.80–18.64)	0.43
**GATA**	Codominant	A/A	56	30.25 (7.24)	*Reference*	0.25
		A/G	40	47.25 (10.37)	18.19 (−5.68–42.05)	
		G/G	4	55.5 (52.5)	31.75 (−28.87–92.37)	
	Dominant	A/A	56	30.25 (7.24)	*Reference*	0.1
		A/G-G/G	44	48 (10.04)	19.44 (−3.54–42.41)	
	Recessive	A/A-A/G	96	37.33 (6.1)	*Reference*	0.43
		G/G	4	55.5 (52.5)	24.77 (−35.95–85.49)	
	Log-additive	---	---	---	17.35 (−2.51–37.20)	0.094

Data presented as mean of Gensini score (standard error of the mean). OR (95% CI), odds ratio (95% confidence interval). The bold values indicate statistical significance, *p* < 0.05.

## Data Availability

All generated data in this study are included in the article.
